# The Shoulder Arthroplasty Multicenter Registry for Advanced Investigation Database (SAMURAI-DB): Design and Structure of a Multicenter Shoulder Arthroplasty Registry in Japan

**DOI:** 10.7759/cureus.117893

**Published:** 2026-10-14

**Authors:** Naoya Kubota, Yoshihiro Hirakawa, Tomoya Manaka, Katsumasa Nakazawa, Yoichi Ito, So Taniguchi, Ayako Ogura, Yuki Takahashi, Yuki Hasegawa, Hidetomi Terai

**Affiliations:** 1 Department of Orthopedic Surgery, Ishikiriseiki Hospital, Higashiōsaka, JPN; 2 Department of Orthopedic Surgery, Graduate School of Medicine, Osaka Metropolitan University, Osaka, JPN; 3 Department of Orthopedic Surgery, Ito Clinic, Osaka Shoulder Center, Matsubara, JPN; 4 Department of Rehabilitation Medicine, Ishikiriseiki Hospital, Higashiōsaka, JPN

**Keywords:** anatomic total shoulder arthroplasty, database, epidemiology, japanese population, large database, redcap, registry, reverse shoulder arthroplasty, reverse total shoulder arthroplasty, shoulder functions

## Abstract

Background: Shoulder arthroplasty has become an increasingly important treatment option for a broad range of shoulder disorders. Although several national shoulder arthroplasty registries have been established in North America, Europe, and Australia, there is currently no well-established multicenter shoulder arthroplasty registry in Japan. To address this unmet need, researchers established the Shoulder Arthroplasty Multicenter Registry for Advanced Investigation Database (SAMURAI-DB), a multicenter clinical registry in Japan. The registry was initially launched in 2014 using paper-based case report forms and was subsequently integrated into the Research Electronic Data Capture (REDCap; Nashville, TN: Vanderbilt University) platform in 2018. The purpose of this study was to describe the design, structure, and data-collection system of SAMURAI-DB and to summarize the characteristics of procedures registered from April 2014 through December 2025.

Methods: The SAMURAI-DB is a multicenter observational registry that prospectively collects clinical, surgical, radiographic, and patient-reported outcome measures (PROMs) data from patients undergoing shoulder arthroplasty. This descriptive analysis included cases registered between April 1, 2014, and December 31, 2025. Data collected included patient demographics, diagnosis, surgical procedure, implant information, complications, radiographic assessments, PROMs, and follow-up status. Registry characteristics and descriptive statistics were analyzed.

Results: A total of 2,115 shoulder arthroplasties from 20 participating institutions were included in the registry. Reverse total shoulder arthroplasty (rTSA) accounted for 1,616 procedures (76.4%), followed by anatomic total shoulder arthroplasty (aTSA) (334 procedures, 15.8%) and revision rTSA (129 procedures, 6.1%). The mean age of patients undergoing rTSA was 76.9 years. The most common indications for rTSA were cuff tear arthropathy (35.1%), irreparable rotator cuff tear (31.3%), proximal humeral fracture (8.8%), and primary osteoarthritis (8.5%). Among all registered procedures eligible for each follow-up interval, outpatient visits were conducted, clinical scores were evaluated, and data were entered into the SAMURAI-DB for 70.3% of eligible procedures at six months (1,353/1,924), 76.6% at one year (1,336/1,744), and 64.8% at two years (939/1,450). Among 1,616 rTSA procedures, the most frequently recorded complications were acromial or scapular spine fracture (3.0%), instability (1.8%), implant loosening (1.6%), and periprosthetic joint infection (1.3%).

Conclusions: SAMURAI-DB represents one of the first large-scale multicenter shoulder arthroplasty registries in Japan utilizing the REDCap platform. The registry enables standardized collection of longitudinal clinical, radiographic, and PROMs data across multiple institutions. The registry provides infrastructure for epidemiological research, future implant surveillance, quality improvement, and future international collaboration in shoulder arthroplasty.

## Introduction

Shoulder arthroplasty has evolved substantially over the past three decades and is now widely accepted as an effective treatment for a broad spectrum of shoulder disorders, including cuff tear arthropathy, primary glenohumeral osteoarthritis, proximal humeral fractures, inflammatory arthritis, and failed previous shoulder arthroplasty [1]. In particular, the indications for reverse total shoulder arthroplasty (rTSA) have expanded considerably since the introduction of the Grammont prosthesis, resulting in a rapid increase in the annual number of procedures performed worldwide.

Large clinical registries have become increasingly important in evaluating the real-world performance of joint arthroplasty. Unlike randomized controlled trials, which typically include selected patient populations with relatively short follow-up periods, arthroplasty registries provide comprehensive information regarding implant utilization, patient demographics, complication profiles, revision surgery, and long-term survivorship in routine clinical practice. Registry-based studies have contributed substantially to improvements in implant design, surgical techniques, and evidence-based decision-making [2].

Several national shoulder arthroplasty registries have been established in recent years [3]. These registries have generated valuable epidemiological data and have facilitated continuous monitoring of implant performance and clinical outcomes. In Japan, shoulder arthroplasty procedures have been included in the Japanese Orthopaedic Association National Registry (JOANR) since 2020 [4]. Nationwide trends in rTSA volume, including data from the period before and after this transition, have also been reported separately [5]. However, detailed multicenter clinical registries specifically dedicated to shoulder arthroplasty remain limited, particularly with respect to longitudinal radiographic evaluation, patient-reported outcome measures (PROMs), and comprehensive perioperative data collection.

To address this unmet need, the Shoulder Arthroplasty Multicenter Registry for Advanced Investigation Database (SAMURAI-DB) was initiated in 2014 as a multicenter collaborative registry for shoulder arthroplasty. In 2018, the registry was migrated to the Research Electronic Data Capture (REDCap; Nashville, TN: Vanderbilt University) platform [6,7]. Data were collected prospectively using paper case report forms from 2014 to 2018 and subsequently transcribed into REDCap; from August 2018 onward, data were entered prospectively directly into REDCap. The registry was designed to provide a comprehensive platform for collecting demographic information, surgical details, implant characteristics, radiographic findings, PROMs, complications, and longitudinal follow-up data.

## Materials and methods

Study design

SAMURAI-DB is an investigator-initiated, multicenter observational registry established to prospectively collect comprehensive clinical data on shoulder arthroplasty procedures in Japan. The registry was designed to evaluate patient demographics, indications for surgery, implant utilization, perioperative complications, radiographic outcomes, PROMs, and long-term implant survivorship.

The registry was initiated on January 1, 2014, as a paper-based multicenter registry. On August 30, 2018, the registry was migrated to the REDCap platform (REDCap; Nashville, TN: Vanderbilt University). Clinical data that had previously been collected using paper case report forms were retrospectively entered into the REDCap database, after which all participating institutions prospectively registered consecutive shoulder arthroplasty procedures using standardized electronic case report forms (Figure 1).

**Figure 1:**
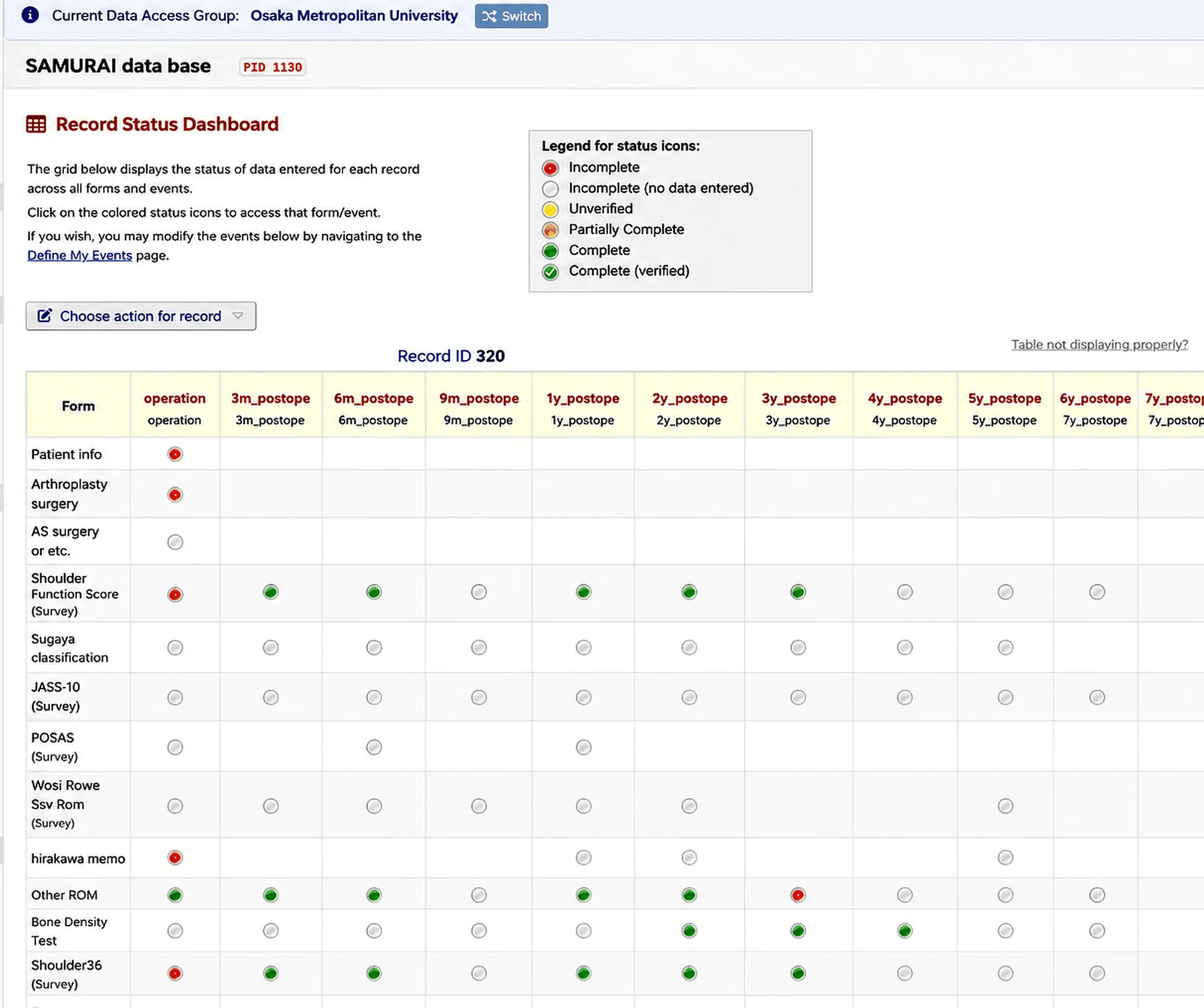
Overview of the SAMURAI-DB in REDCap. The screenshot shows the record display interface of the SAMURAI-DB. Longitudinal data collection is organized by predefined postoperative time points, including the preoperative period and follow-up at three months, six months, nine months, one year, two years, and subsequent annual intervals. The prespecified assessment windows were four to eight months for the six-month assessment, nine to 15 months for the one-year assessment, 21-27 months for the two-year assessment, and 33-39 months for the three-year assessment. An assessment was considered completed only when it was performed within the corresponding assessment window. Assessments performed outside these windows were retained in the registry but were not counted as completed assessments for the respective scheduled time point. The system is highly customizable, allowing users to freely add data fields and assessment items according to their specific needs. This flexibility allows SAMURAI-DB to accommodate evolving data-collection needs across participating institutions. Screenshots of the Research Electronic Data Capture (REDCap) platform, developed by Vanderbilt University, are reproduced with permission. SAMURAI-DB: Shoulder Arthroplasty Multicenter Registry for Advanced Investigation Database; REDCap: Research Electronic Data Capture; 3m: three months; 6m: six months; 9m: nine months; 1y: one year; 2y: two years; 3y: three years; 4y: four years; 5y: five years; 6y: six years; AS: arthroscopic surgery; postope: postoperation; JASS-10: Joint Awareness Score for Shoulder-10; POSAS: Patient and Observer Scar Assessment Scale; WOSI: Western Ontario Shoulder Instability Index; SSV: subjective shoulder value; ROM: range of motion

This study describes the design, structure, and baseline characteristics of the registry and includes all eligible shoulder arthroplasty procedures between April 1, 2014, and December 31, 2025. SAMURAI-DB is coordinated by Osaka Metropolitan University and currently includes 20 participating institutions distributed throughout Japan. Participating hospitals include university hospitals, regional referral centers, and high-volume orthopedic hospitals specialized in shoulder surgery. Each participating institution is responsible for prospective procedure registration and follow-up using standardized data collection protocols. Data were entered directly into the REDCap system through secure web-based access by authorized investigators.

All shoulder arthroplasty procedures performed at participating institutions during the study period were considered eligible for registration. Participating institutions were asked to register consecutive procedures. The registry captures the following procedure categories: primary rTSA, primary anatomic total shoulder arthroplasty (aTSA), primary hemiarthroplasty (HA), revision rTSA, revision aTSA, revision HA, implant removal, and other shoulder arthroplasty procedures. Revision rTSA is defined as conversion of any prior arthroplasty to an rTSA construct. To improve transparency, SAMURAI-DB will distinguish the original index arthroplasty from subsequent procedures. A primary arthroplasty is the first shoulder arthroplasty performed on a shoulder. A revision arthroplasty is a subsequent procedure involving modification, addition, exchange, or removal of one or more prosthetic components of a previous arthroplasty. A conversion is a change from one arthroplasty construct to another (for example, aTSA to rTSA or HA to rTSA) and is attributed as a revision event to the original arthroplasty that was converted; the newly implanted construct is not counted as a new primary arthroplasty. Revision without conversion refers to component exchange or modification while retaining the same overall arthroplasty construct. Reoperation without revision refers to a subsequent procedure that does not modify or remove the arthroplasty components. Explant/removal without preimplantation refers to prosthesis removal without immediate replacement. Second-stage reimplantation refers to reimplantation following staged removal. Open reduction and internal fixation (ORIF) followed by rTSA is not classified as revision arthroplasty because ORIF is not an arthroplasty; instead, the rTSA is recorded as the index arthroplasty with prior ORIF documented as previous shoulder surgery. Each subsequent procedure is linked to the shoulder, laterality, and relevant index arthroplasty.

Postoperative complications were defined as adverse events occurring after the index arthroplasty that were considered clinically relevant and related to the surgical procedure or implant. Subsequent revision or reoperation procedures were recorded separately from the complication event and were not considered synonymous with complications. Postoperative complications were recorded descriptively in the registry. Because follow-up duration varied among patients and longitudinal complication adjudication and event-date recording were not uniformly standardized across participating institutions, complication frequencies were calculated as crude proportions among registered procedures and were not interpreted as time-to-event incidence rates.

Clinical data were collected using standardized electronic case report forms developed specifically for SAMURAI-DB. The registry currently consists of 24 major data domains comprising 481 individual variables. Data are collected longitudinally from the preoperative assessment through postoperative follow-up for up to 15 years. The major data domains include patient demographics, medical history, surgical information, implant characteristics, complications, radiographic evaluation, revision surgery, clinical outcomes, and PROMs. All participating investigators received standardized instructions regarding data entry to ensure consistency across institutions.

The REDCap database incorporates predefined data fields, standardized variable definitions, branching logic, audit trails, user authentication, and role-based access control. These functions facilitate standardized data collection while minimizing entry errors and maintaining data security. The longitudinal design of REDCap allows repeated assessments at predefined postoperative intervals while preserving complete historical records for each procedure.

Baseline demographic information includes age, sex, body mass index, affected side, diagnosis, comorbidities, smoking history, and previous shoulder surgery. Operative variables include surgical procedure, implant manufacturer, implant model, fixation technique, glenoid component characteristics, humeral stem characteristics, polyethylene insert thickness, glenosphere size, surgical approach, operative time, estimated blood loss, and intraoperative complications. Clinical outcomes included shoulder range of motion, pain assessed using the visual analog scale (VAS), and the Constant score, all of which were collected prospectively at predefined follow-up intervals. The registry prospectively collects PROMs before surgery and during postoperative follow-up. PROMs currently include the American Shoulder and Elbow Surgeons (ASES) score and the subjective shoulder value (SSV). Procedures are evaluated preoperatively and postoperatively at six months, one year, two years, three years, five years, 10 years, and 15 years after surgery (Figure 2).

**Figure 2:**
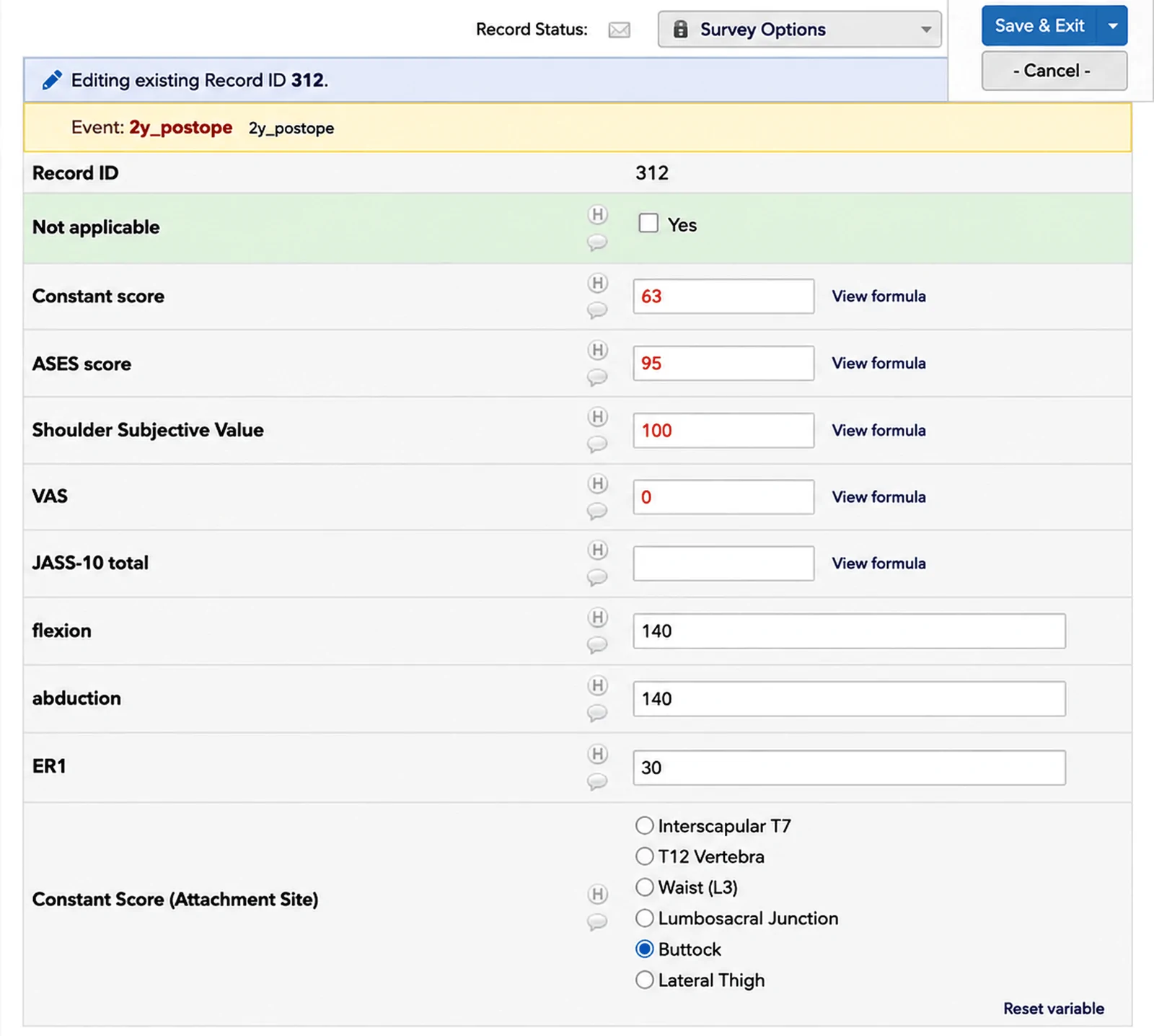
Clinical assessment page of the SAMURAI-DB using REDCap. The system enables automatic calculation and storage of clinical outcome scores based on predefined questionnaires and physical examination data entered at each follow-up visit. This screenshot shows the clinical assessment at two years postoperatively. By entering the range of motion measurements and responses to the predefined questionnaires, each clinical outcome score is automatically calculated and stored in the database. Calculated scores are automatically stored in the database, supporting standardized longitudinal data accumulation. Screenshots of the Research Electronic Data Capture (REDCap) platform, developed by Vanderbilt University, are reproduced with permission. SAMURAI-DB: Shoulder Arthroplasty Multicenter Registry for Advanced Investigation Database; REDCap: Research Electronic Data Capture; ASES: American Shoulder and Elbow Surgeons; VAS: visual analog scale; JASS-10: Joint Awareness Score for Shoulder-10; ER1: external rotation with the arm at the side

Patients were encouraged by the treating physicians to attend postoperative visits at the predefined follow-up time points, and a designated staff member additionally facilitated follow-up attendance. Patient-specific preoperative characteristics, operative details, and postoperative complications are recorded by the treating physicians. Clinical outcome measures and PROMs are assessed by designated physical therapists according to the predefined assessment protocol. The collected data are subsequently entered into the REDCap database. This standardized division of responsibilities is intended to facilitate consistent longitudinal data collection across participating institutions.

Eligible follow-up rates were calculated by including in the denominator only those procedures for which the scheduled follow-up interval had elapsed by December 31, 2025. A procedure was considered to have achieved follow-up at a given time point if follow-up data were recorded within the corresponding assessment window. The primary objective of the present study was to describe the structure and characteristics of SAMURAI-DB. Participating institutions, the number of registered procedures, patient demographics, surgical indications, procedure distribution, follow-up rates, and complication profiles were described. No comparative analyses between treatment groups were planned because the purpose of this study was descriptive.

## Results

Between April 1, 2014, and December 31, 2025, a total of 1,959 patients and 2,115 procedures were registered in the SAMURAI-DB. During the study period, the registry expanded to include 20 participating institutions, consisting of university hospitals, regional referral centers, and specialized orthopedic hospitals in Japan. Among the participating institutions, Ito Clinic contributed the largest proportion of registered cases (53.9%), followed by Osaka Metropolitan University Hospital (22.6%) and Ishikiriseiki Hospital (6.6%).

Data completeness at baseline was high for core registry variables as follows: sex 99.8% (2,110/2,115); age 99.4% (2,103/2,115); diagnosis 99.5% (2,105/2,115); procedure type 100% (2,115/2,115); and surgical approach 99.3% (2,100/2,115). Baseline American Shoulder and Elbow Surgeons (ASES) score and subjective shoulder value (SSV) were available in 59.1% (1,251/2,115) and 59.5% (1,258/2,115) of procedures, respectively; either ASES score or SSV was available in 66.4% (1,404/2,115).

A total of 2,115 shoulder arthroplasty procedures were available for analysis. Primary rTSA accounted for the majority of procedures (1,616 procedures; 76.4%). Other procedures included aTSA in 334 procedures (15.8%), revision rTSA in 129 procedures (6.1%), hemiarthroplasty (HA) in 19 procedures (0.9%), revision HA in five procedures (0.2%), revision aTSA in two procedures (0.1%), implant removal in five procedures (0.2%), and other procedures in five procedures (0.2%) (Table 1).

**Table 1:** The distribution of the 2,115 shoulder arthroplasty procedures registered in SAMURAI-DB between April 2014 and December 2025, categorized by procedure type. rTSA: reverse total shoulder arthroplasty; aTSA: anatomic total shoulder arthroplasty; HA: hemiarthroplasty; SAMURAI-DB: Shoulder Arthroplasty Multicenter Registry for Advanced Investigation Database

Procedures	Number	Percentage
rTSA	1,616	76.4
aTSA	334	15.8
Revision rTSA	129	6.1
HA	19	0.9
Implant removal	5	0.2
Revision HA	5	0.2
Revision aTSA	2	0.1
Other	5	0.2

The annual number of registered rTSA procedures increased progressively throughout the study period. The annual mean age of patients undergoing rTSA ranged from 76.0 to 77.8 years (Table 2). Among rTSA procedures, the most common indication was cuff tear arthropathy (CTA), accounting for 568 procedures (35.1%). Irreparable rotator cuff tear (RCT) was the second most common indication, with 505 procedures (31.3%), followed by proximal humeral fracture (PHF) in 142 procedures (8.8%) and primary osteoarthritis (OA) in 138 procedures (8.5%). Other indications included chronic dislocation, combined osteoarthritis with irreparable rotator cuff tear, rheumatoid arthritis, and miscellaneous disorders (Table 3).

**Table 2:** Annual number of rTSA procedures and mean patient age for each year. rTSA: reverse total shoulder arthroplasty

Year	Number of rTSA	Mean patient age
2014	36	76.8
2015	49	76.0
2016	55	76.9
2017	94	77.0
2018	106	76.5
2019	137	76.4
2020	131	76.3
2021	132	77.7
2022	163	77.1
2023	189	77.2
2024	230	77.8
2025	294	76.6

**Table 3:** Indications for rTSA. The OA+RCT category is limited to cases with rotator cuff tears, up to and including large tears. Massive rotator cuff tears are classified as RCT or CTA, depending on the presence of cuff tear arthropathy. CTA: cuff tear arthropathy; RCT: irreparable rotator cuff tear; PHF: proximal humeral fracture; OA: osteoarthritis; RA: rheumatoid arthritis; rTSA: reverse total shoulder arthroplasty

Indications	Number	Percentage
CTA	568	35.1
RCT	505	31.3
PHF	142	8.8
OA	138	8.5
Fracture sequelae	96	5.9
Chronic dislocation	71	4.4
OA+RCT	46	2.8
RA	14	0.9
Others	36	2.2

Follow-up completeness was evaluated using the eligible follow-up rate. For each scheduled follow-up interval, the denominator included only procedures for which the relevant postoperative time had elapsed by December 31, 2025. Follow-up rates were calculated across all registered shoulder arthroplasty procedures, not limited to a specific procedure type. The eligible follow-up rate for clinical assessment based on the Constant score was available for 70.3% at six months, 76.6% at one year, and 64.8% at two years. Either ASES or SSV was available for 45.2%, 51.6%, and 42.9%, respectively (Table 4).

**Table 4:** Eligible follow-up rate. ASES: American Shoulder and Elbow Surgeons score; SSV: subjective shoulder value

Timepoint	Eligible	Clinical, n (%)	ASES, n (%)	SSV, n (%)	Either score, n (%)
6 months	1,924	1,353 (70.3)	821 (42.7)	791 (41.1)	870 (45.2)
1 year	1,744	1,336 (76.6)	779 (44.7)	846 (48.5)	900 (51.6)
2 years	1,450	939 (64.8)	525 (36.2)	600 (41.4)	622 (42.9)

Complications

Among the 1,616 primary rTSA procedures, the most frequently recorded postoperative complication was acromial or scapular spine fracture, observed in 49 procedures (3.0%). Instability was reported in 29 procedures (1.8%), implant loosening in 26 procedures (1.6%), and periprosthetic infection in 21 procedures (1.3%). No other complication exceeded a frequency of 1% (Table 5).

**Table 5:** Complications of rTSA. rTSA: reverse total shoulder arthroplasty

Complications	Number	Frequency (%)
Acromial or scapular spine fracture	49	3.0
Instability	29	1.8
Implant loosening	26	1.6
Infection	21	1.3
Nerve palsy	16	1.0
Periprosthetic fracture	15	0.9

## Discussion

This study describes the establishment, design, and current status of SAMURAI-DB, a multicenter shoulder arthroplasty registry in Japan that uses the REDCap platform to capture and manage data. Since its initiation in 2014 and subsequent transition from a paper-based registry to REDCap in 2018, the registry has prospectively collected standardized clinical data from 20 institutions and has accumulated more than 2,100 shoulder arthroplasty procedures. SAMURAI-DB integrates patient demographics, surgical details, implant characteristics, radiographic assessments, patient-reported outcome measures, and postoperative complications into a single longitudinal database across multiple participating institutions in Japan.

Clinical registries have become an indispensable component of modern arthroplasty research because the true performance of joint replacement implants cannot be fully evaluated by randomized controlled trials (RCTs) alone [8]. Implant designs continue to evolve, surgical techniques are constantly refined, and patient characteristics differ substantially among institutions and countries. Consequently, long-term implant performance in routine clinical practice cannot be adequately assessed by RCTs, which generally involve relatively small sample sizes, strict inclusion criteria, and limited follow-up durations. Arthroplasty registries have three objectives. First, registries accurately define the epidemiology of arthroplasty within a specific population. Second, they provide timely information regarding surgical outcomes to the orthopedic community. Third, they identify factors associated with poor outcomes and provide continuous feedback to participating institutions and surgeons, ultimately improving the quality of patient care [2,9]. Previous studies comparing registry-based evidence with data derived from peer-reviewed clinical studies have demonstrated comparable implant failure rates, indicating that registry data provide reliable evidence while allowing earlier recognition of changes in clinical practice and implant performance [10,11].

National arthroplasty registries were first introduced in Sweden in 1975 and subsequently expanded throughout Scandinavia before being adopted across Europe, Oceania, and North America. The Swedish Hip Arthroplasty Register, established in 1979, has enabled hospitals to benchmark outcomes against national data and has contributed substantially to early identification of poorly performing implants [12,13]. National registries for total hip arthroplasty (THA) and total knee arthroplasty (TKA) have been successfully implemented in many countries and have substantially improved implant surveillance and quality assurance [3,14-17]. Similarly, nationwide arthroplasty registries in Japan have gradually expanded since 2003, primarily focusing on hip and knee arthroplasty [4]. Similarly, national registries dedicated to shoulder arthroplasty have expanded rapidly since the introduction of the Grammont rTSA prosthesis in 1991 [1,18-21].

Shoulder arthroplasty registry activity in Japan has expanded since 2014, when rTSA was introduced into clinical practice. According to a nationwide database analysis, the annual number of rTSA procedures performed in Japan increased from 630 in 2014 to 5,601 in 2023, accounting for the majority of the growth in shoulder arthroplasty volume [5]. These figures were derived from a nationwide database analysis covering the period before and after the establishment of the current Japanese Orthopaedic Association National Registry (JOANR); until 2019, rTSA cases in Japan were primarily captured through the registry system operated by the Japanese Society for Replacement Arthroplasty, and from 2020 onward, shoulder arthroplasty procedures have been included in the JOANR, as noted in the Introduction. In Japan, where the number of rTSA procedures is rapidly increasing, registry data provide information on procedure volume, indications, and regional distribution of surgery. However, nationwide registries generally do not collect detailed clinical outcome measures such as the ASES score, Japanese Orthopaedic Association (JOA) score, University of California, Los Angeles (UCLA) score, or shoulder range of motion. In addition, annual reporting inevitably introduces a delay between clinical practice and data dissemination [22]. SAMURAI-DB serves a different role - it is a voluntary multicenter clinical registry that captures more detailed clinical, implant, radiographic, and PROM information. This complementary architecture may support research questions that cannot be answered using procedure-volume data alone. Collection of PROM data has the potential to inform value-based care, quality assurance and improvement initiatives, refinement of surgical indications, and shared decision-making regarding surgical timing and treatment endpoints [23-26].

Although nationwide registries provide larger sample sizes, multicenter clinical registries dedicated to shoulder arthroplasty remain uncommon. The Scottish Shoulder Arthroplasty Registry is one representative example in which 20 shoulder surgeons voluntarily registered demographic information, implant characteristics, surgical procedures, perioperative complications, postoperative pain, activity level, and patient satisfaction [27]. Although national registries benefit from large sample sizes and broad population coverage, voluntary multicenter clinical registries may offer advantages in data granularity, follow-up completeness, and the depth of clinical and radiographic assessment [28]. The eligible follow-up rates of clinical outcomes in SAMURAI-DB were 70.3% at six months, 76.6% at one year, and 64.8% at two years. These rates may be considered acceptable for a voluntary multicenter clinical registry. The REDCap platform provides several advantages for the management of multicenter clinical registries. Standardized electronic case report forms, predefined data fields, branching logic, and mandatory fields reduce data entry errors and improve data completeness [6,7]. In addition, audit trails, user authentication, and role-based access control enhance data security and ensure traceability of all modifications. The primary strength of SAMURAI-DB is its relatively granular clinical and procedural data structure, which enables characterization of patient demographics, surgical indications, procedure types, implant characteristics, clinical outcomes, and PROMs across participating institutions. The registry also links repeated procedure-specific records and longitudinal follow-up assessments, providing a foundation for evaluating changes in clinical outcomes and implant utilization over time. At its current stage, SAMURAI-DB can reliably support descriptive analyses of real-world shoulder arthroplasty practice, including procedure trends, implant utilization, patient characteristics, and patterns of postoperative follow-up.

However, several components require further development before the registry can reliably support definitive implant survivorship or longitudinal outcome studies. Follow-up retention decreases at longer time points, and PROM completion is lower than clinical follow-up completion. In addition, radiographic follow-up is currently performed mainly for selected implant types or individual research projects and is not yet standardized across the entire registry. Complication adjudication and event-date recording also require further standardization. Furthermore, the current registry has substantial site imbalance, with a large proportion of procedures contributed by a small number of institutions, which may limit the generalizability of aggregated results.

Future infrastructure development should therefore focus on improving longitudinal patient and procedure linkage; standardizing definitions and recording of complications and revision events; systematically recording event dates; standardizing radiographic assessments; improving PRO completion and long-term retention; and formalizing data validation and auditing procedures. These developments would strengthen the ability of SAMURAI-DB to perform time-to-event analyses, evaluate implant survivorship, and investigate longitudinal clinical and patient-reported outcomes.

This study has several limitations. First, SAMURAI-DB is a voluntary multicenter registry with non-population-based enrollment, and the 20 participating institutions do not provide comprehensive national coverage. Accordingly, the procedure distribution and patient characteristics reported in this study reflect the composition of the registry rather than nationwide shoulder arthroplasty practice in Japan. Second, a single institution (Ito Clinic) contributed 53.9% of the registered cases, which may have introduced site-level bias into the aggregated demographic and outcome data. Third, the transition from paper-based to electronic data collection in 2018 may have resulted in differences in data completeness between the two collection periods. Fourth, the proportion of patients eligible for follow-up decreased at longer follow-up intervals, and complication frequencies may therefore be underestimated among patients without long-term follow-up. Fifth, complication proportions were calculated without adjustment for differences in the duration of observation, limiting their interpretation as true event rates. Sixth, surgical techniques, implant selection, and postoperative protocols were not standardized across participating institutions. Seventh, although radiographic assessments are available in SAMURAI-DB, longitudinal radiographic follow-up is currently performed mainly for specific implant types or individual research projects and is not yet standardized across the entire registry. Therefore, radiographic follow-up completeness was not included in the present analysis. Eighth, mortality was not specifically assessed in the present study, and standardized mortality or vital-status linkage was not available in the current registry. Ninth, PROM completion was substantially lower than clinical follow-up completion. This incomplete PROM capture may introduce attrition and response bias and limit the representativeness of longitudinal PROM analyses. Finally, no formal data validation or audit procedures have been described.

## Conclusions

This study describes the design, structure, and current status of SAMURAI-DB, a multicenter shoulder arthroplasty registry established in Japan in 2014 and migrated to the REDCap platform in 2018. As of December 31, 2025, the registry included 1,959 patients and 2,115 procedures from 20 participating institutions, with 24 major data domains and 481 individual variables covering patient characteristics, surgical procedures, implant information, clinical outcomes, PROMs, radiographic assessments, complications, and subsequent procedures.

SAMURAI-DB provides a relatively granular platform for describing real-world shoulder arthroplasty practice, including patient characteristics, surgical procedures, implant utilization, and patterns of postoperative clinical and PROM follow-up. However, incomplete long-term follow-up and PROM capture, substantial variation in institutional contributions, and the lack of standardized longitudinal radiographic assessment, complication adjudication, and event-date recording currently limit its use for definitive implant survivorship and longitudinal outcome analyses. Continued development of standardized patient- and procedure-level longitudinal linkage, radiographic assessment, PROM collection, complication and revision-event recording, and formal data validation will be important to strengthen the registry. These improvements will enhance the capacity of SAMURAI-DB to support future longitudinal implant surveillance, time-to-event analyses, and clinical outcome research in shoulder arthroplasty.
